# Supplementary material for: Host environment shapes filarial parasite fitness and Wolbachia endosymbionts dynamics
Source: PLoS Pathog. 2025 Jul 11;21(7):e1013301. doi: 10.1371/journal.ppat.1013301 (PMC12270307; doi:10.1371/journal.ppat.1013301)
Supplement: S1 Fig — Type 2-competent wild-type (WT) and type 2-deficient (Il4rα-/-/Il5-/-, KO) mice were inoculated with 40 infective larvae (L3) of the filaria L. sigmodontis. Parasites were harvested and measured at various time points before and after the fourth molt (~30 dpi), and Wolbachia’s gene ftsZ and filarial actin were evaluated by qPCR in female filariae. (A) Measurements of worm length in millimeters (mm) from 24 to 70 days post-infection (dpi) in wild-type and type 2-deficient (Il4rα-/-/Il5-/-) mice. Results are expressed as the mean ± SD of n = 20–40 filariae per group (24–50 dpi), and n = 8 filariae from wild-type and 28 filariae from Il4rα-/-/Il5-/- hosts (60 dpi). Two-way ANOVAs followed by Bonferroni’s multiple comparisons tests were performed; ***p < 0.001, ****p < 0.0001 indicate significant difference between filariae from wild-type and Il4rα-/-/Il5-/- hosts. (B) Quantification of actin gene expression, normalized to worm length, over the same period. These measurements provide a baseline for the relative quantification of Wolbachia density shown in Fig 1, accounting for changes in worm size that could influence the interpretation of bacterial load. Results are expressed as the mean ± SD of n = 4–6 filariae per group (24–60 dpi), n = 11–15 filariae per group (70 dpi). Two-way ANOVAs followed by Bonferroni’s multiple comparisons tests were performed; **p < 0.01, ***p < 0.001 indicate significant difference between filariae from wild-type and Il4rα-/-/Il5-/- hosts. (PDF) [file ppat.1013301.s001.pdf]

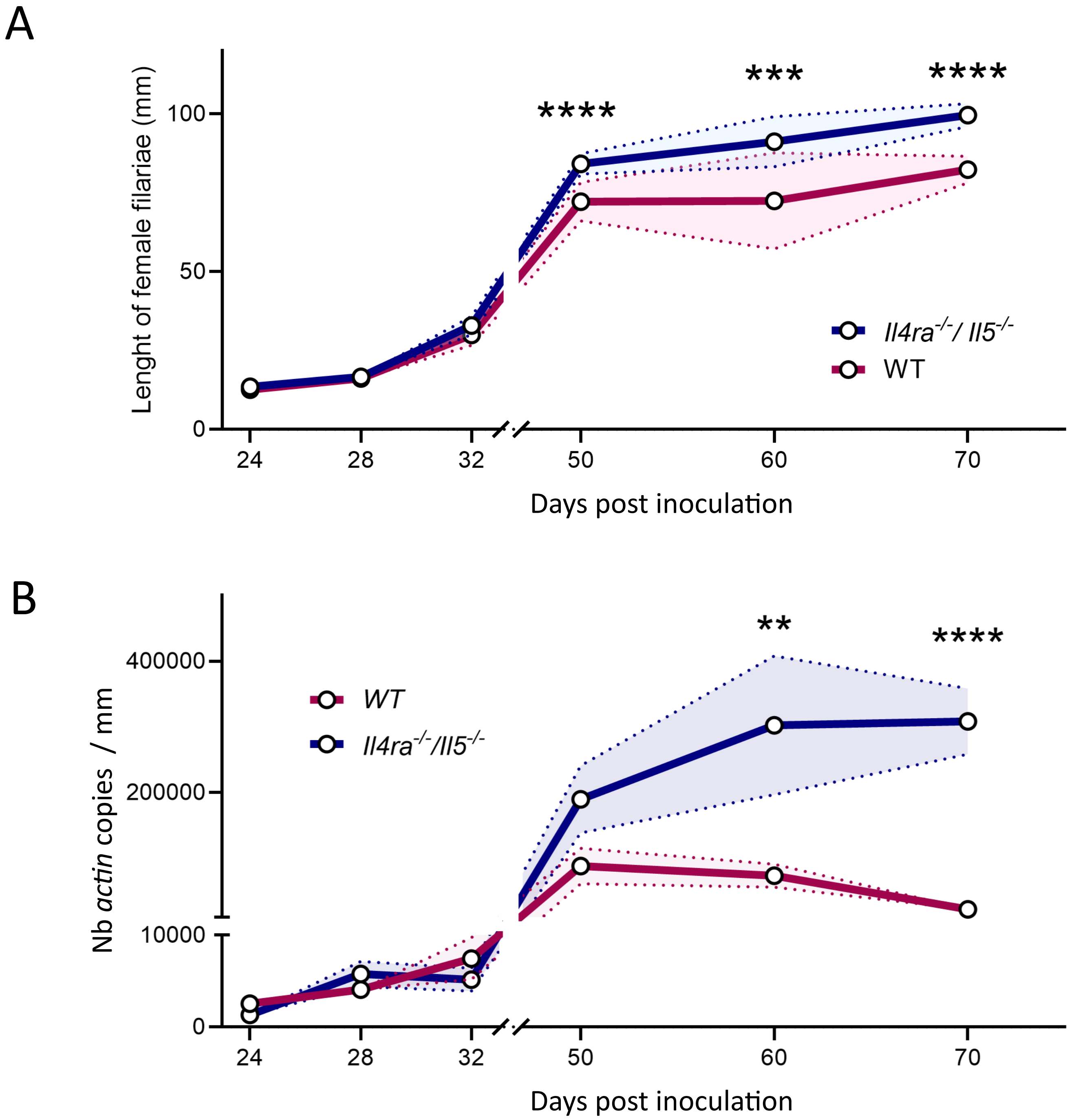

Supplementary Figure 1: Growth and *actin* gene expression of female *Litomosoides sigmodontis* in wild-type and type 2-deficient mice. Type 2-competent wild-type (WT) and type 2-deficient (*Il4rα*$^{-/-}$*/Il5*$^{-/-}$*, KO*) mice were inoculated with 40 infective larvae (L3) of the filaria *L. sigmodontis*. Parasites were harvested and measured at various time points before and after the fourth molt (~30 dpi), and *Wolbachia's* gene *ftsZ* and filarial *actin* were evaluated by qPCR in female filariae. **(A)** Measurements of worm length in millimeters (mm) from 24 to 70 days post-infection (dpi) in wild-type and type 2-deficient (*Il4rα*$^{-/-}$*/Il5*$^{-/-}$) mice. Results are expressed as the mean ± SD of n= 20-40 filariae per group (24-50 dpi), and n = 8 filariae from wild-type and 28 filariae from *Il4rα*$^{-/-}$*/Il5*$^{-/-}$ hosts (60 dpi) . Two-way ANOVAs followed by Bonferroni's multiple comparisons tests were performed; ***p < 0.001, ****p < 0.0001 indicate significant difference between filariae from wild-type and *Il4rα*$^{-/-}$*/Il5*$^{-/-}$ hosts. **(B)** Quantification of *actin* gene expression, normalized to worm length, over the same period. These measurements provide a baseline for the relative quantification of *Wolbachia* density shown in Figure 1, accounting for changes in worm size that could influence the interpretation of bacterial load. Results are expressed as the mean ± SD of n = 4-6 filariae per group (24-60 dpi), n = 11-15 filariae per group (70 dpi). Two-way ANOVAs followed by Bonferroni's multiple comparisons tests were performed; **p < 0.01, ***p < 0.001 indicate significant difference between filariae from wild-type and *Il4rα*$^{-/-}$*/Il5*$^{-/-}$ hosts.
