# Supplementary material for: Host environment shapes filarial parasite fitness and Wolbachia endosymbionts dynamics
Source: PLoS Pathog. 2025 Jul 11;21(7):e1013301. doi: 10.1371/journal.ppat.1013301 (PMC12270307; doi:10.1371/journal.ppat.1013301)
Supplement: S2 Fig — (A) Number of filariae recovered in the pleural cavity of WT, KO, and KO + DR mice at 70 dpi. Results are expressed as mean ± SEM, with individual data points representing each mouse (n = 6–8 mice per group). One-way ANOVA followed by Tukey’s multiple comparison test were performed. **p < 0.01, ns: not significant. (B) Female filariae size (cm) from WT, KO, and KO + DR mice at 70 dpi. Results are expressed as mean ± SEM, with individual data points representing each measured worm (n = 6–8 worms per group). One-way ANOVA followed by Tukey’s multiple comparison test were performed. **p < 0.01, ***p < 0.001. (PDF) [file ppat.1013301.s002.pdf]

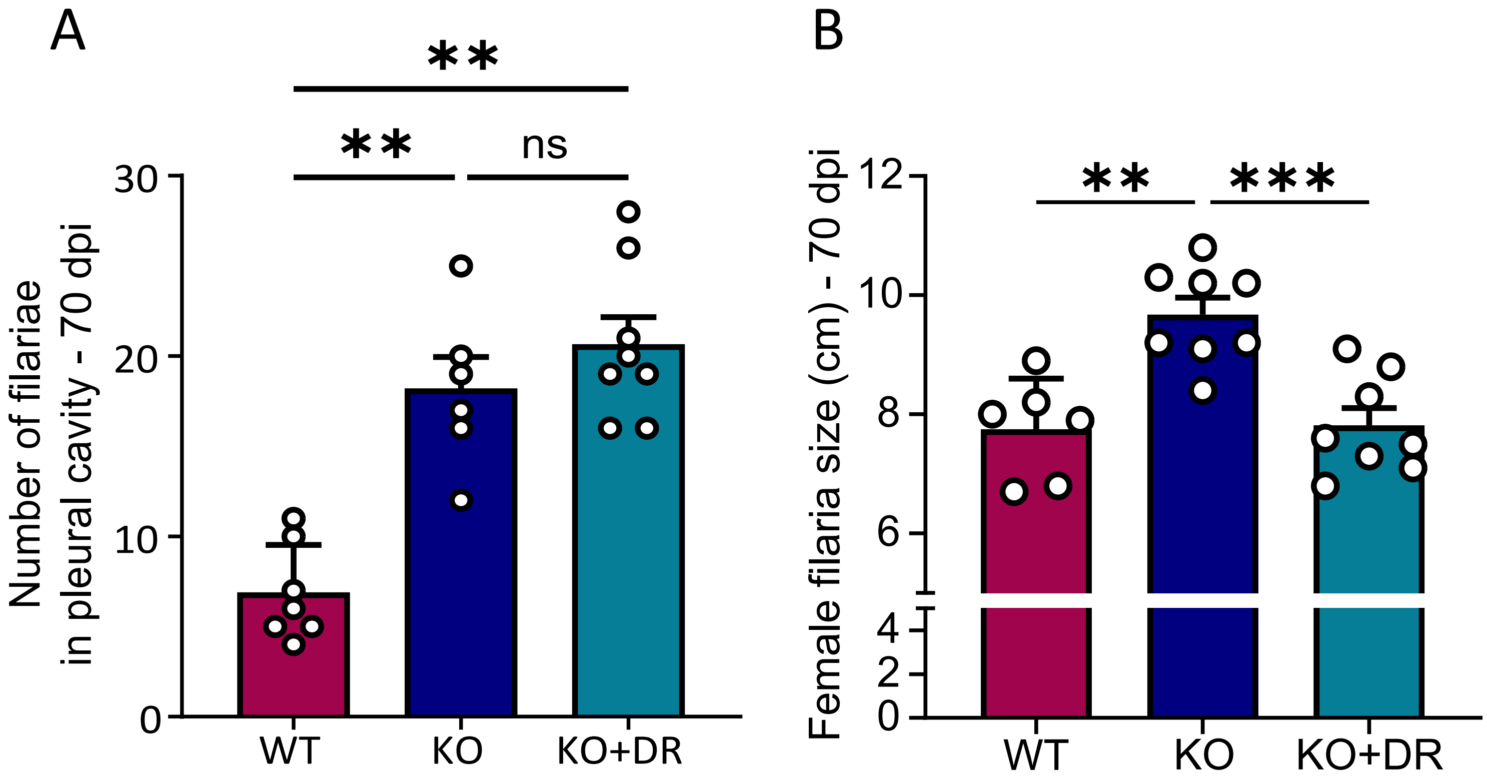

Supplementary Figure 2: Effect of host immune background and *Wolbachia* depletion on filarial size and pleural cavity burden at 70 dpi. (A) Number of filariae recovered in the pleural cavity of WT, KO, and KO+DR mice at 70 dpi. Results are expressed as mean ± SEM, with individual data points representing each mouse (n = 6–8 mice per group). One-way ANOVA followed by Tukey's multiple comparison test were performed. **p < 0.01, ns: not significant.

(B) Female filariae size (cm) from WT, KO, and KO+DR mice at 70 dpi. Results are expressed as mean ± SEM, with individual data points representing each measured worm (n = 6–8 worms per group). One-way ANOVA followed by Tukey's multiple comparison test were performed. **p < 0.01, ***p < 0.001.
