# Supplementary material for: Host environment shapes filarial parasite fitness and Wolbachia endosymbionts dynamics
Source: PLoS Pathog. 2025 Jul 11;21(7):e1013301. doi: 10.1371/journal.ppat.1013301 (PMC12270307; doi:10.1371/journal.ppat.1013301)
Supplement: S3 Fig — (A) Fluorescent microscopy images of an adult female filaria highlighting key anatomical regions. The distal ampulla and ovaries are situated at the posterior end, transitioning through the oviduct into the uteri, which extend along the body axis. The proximal uteri, near to the ovojector, contain mature microfilariae. Staining highlights nuclei (DAPI, blue) and actin filaments (gray). (B) Schematic representation of the female reproductive system. The ovary includes a proliferative zone (PZ, red), where germ cells divide, and a meiotic zone (MZ, blue), where meiosis occurs. The uteri (orange) extend anteriorly and terminate at the ovojector, where mature microfilariae are expelled. (C) Diagram of the distal ovary, showing the rachis (central cytoplasmic core) surrounded by germ cells. Wolbachia (yellow) are distributed along the rachis and within some sheath cells. The ampulla marks the distal tip of the ovary and the starting point of oogenesis. In somatic tissues, Wolbachia also reside in the lateral hypodermal chords (see Fig 2E). (PDF) [file ppat.1013301.s003.pdf]

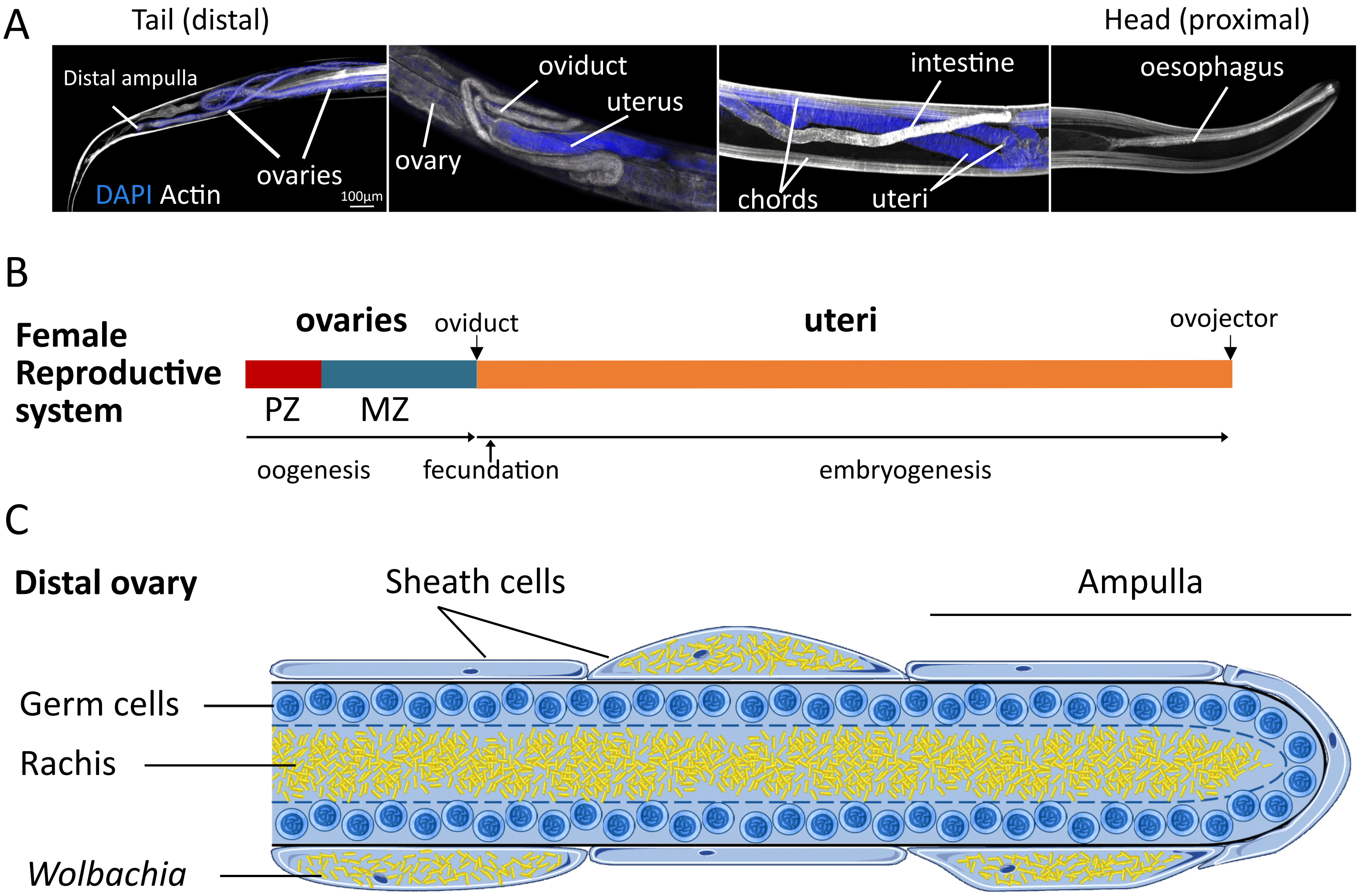

Supplementary Figure 3. Anatomical organization of the female reproductive system and *Wolbachia* localization in *Litomosoides sigmodontis*. (A) Fluorescent microscopy images of an adult female filaria highlighting key anatomical regions. The distal ampulla and ovaries are situated at the posterior end, transitioning through the oviduct into the uteri, which extend along the body axis. The proximal uteri, near to the ovojector, contain mature microfilariae. Staining highlights nuclei (DAPI, blue) and actin filaments (gray). **(B)** Schematic representation of the female reproductive system. The ovary includes a proliferative zone (PZ, red), where germ cells divide, and a meiotic zone (MZ, blue), where meiosis occurs. The uteri (orange) extend anteriorly and terminate at the ovojector, where mature microfilariae are expelled. **(C)** Diagram of the distal ovary, showing the rachis (central cytoplasmic core) surrounded by germ cells. *Wolbachia* (yellow) are distributed along the rachis and within some sheath cells. The ampulla marks the distal tip of the ovary and the starting point of oogenesis. In somatic tissues, *Wolbachia* also reside in the lateral hypodermal chords (see Figure 2E).
