# Supplementary material for: Host environment shapes filarial parasite fitness and Wolbachia endosymbionts dynamics
Source: PLoS Pathog. 2025 Jul 11;21(7):e1013301. doi: 10.1371/journal.ppat.1013301 (PMC12270307; doi:10.1371/journal.ppat.1013301)
Supplement: S4 Fig — (A) Quantification of Wolbachia density (µm2/mm) in Litomosoides sigmodontis Wb(+) larvae at different days post-inoculation, based on fluorescence microscopy images of entire larvae. Brown-Forsythe ANOVA test followed by a Dunnett’s T3 multiple comparisons post-hoc test (***p < 0.001). (B) Confocal fluorescence image of a Wb(+) L4 female L. sigmodontis at day 14 post-infection (D14). The worm was stained for DNA (DAPI, cyan) and Wolbachia (16S ribosomal RNA, yellow). The anterior-to-posterior orientation is indicated (h = head; t = tail). (C) Magnified region showing the lateral hypodermal chords and ovarian primordia in the boxed area from (B). Wolbachia are visible in the lateral chords (Chord1 and Chord2) and are invading the ovarian primordia. (PDF) [file ppat.1013301.s004.pdf]

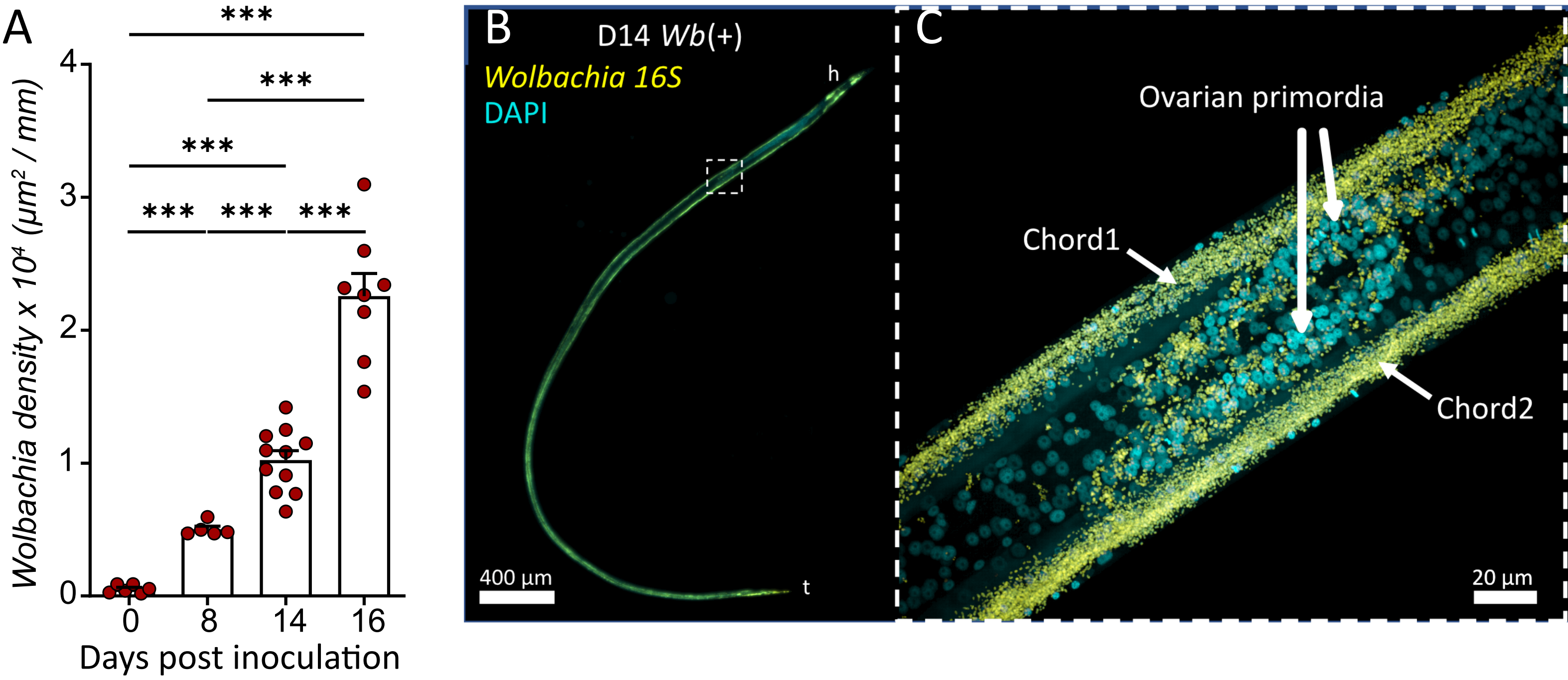

Supplementary Figure 4. Colonization of the ovarian primordia by *Wolbachia* in L4 female filariae. (A) Quantification of *Wolbachia* density (µm²/mm) in *Litomosoides sigmodontis Wb*(+) larvae at different days post-inoculation, based on fluorescence microscopy images of entire larvae. Brown-Forsythe ANOVA test followed by a Dunnett's T3 multiple comparisons post-hoc test (****p* < 0.001). **(B)** Confocal fluorescence image of a *Wb*(+) L4 female *L. sigmodontis* at day 14 post-infection (D14). The worm was stained for DNA (DAPI, cyan) and *Wolbachia* (16S ribosomal RNA, yellow). The anterior-to-posterior orientation is indicated (h = head; t = tail). **(C)** Magnified region showing the lateral hypodermal chords and ovarian primordia in the boxed area from (B). *Wolbachia* are visible in the lateral chords (Chord1 and Chord2) and are invading the ovarian primordia.
